# Repetitive element hypermethylation in multiple sclerosis patients

**DOI:** 10.1186/s12863-016-0395-0

**Published:** 2016-06-18

**Authors:** K. Y. Neven, M. Piola, L. Angelici, F. Cortini, C. Fenoglio, D. Galimberti, A. C. Pesatori, E. Scarpini, V. Bollati

**Affiliations:** Department of Clinical Sciences and Community Health, EPIGET – Epidemiology, Epigenetics and Toxicology Lab, Università degli Studi di Milano, Milan, Italy; Centre for Environmental Sciences, Hasselt University, Diepenbeek, Belgium; Neurology Unit, Saronno ASST Valle Olona Hospital, Saronno, Italy; Department of Preventive Medicine, Fondazione IRCCS Ca’ Granda Ospedale Maggiore Policlinico, Epidemiology Unit, Milan, Italy; Department of Pathophysiology and Transplantation, Dino Ferrari Centre, Università degli Studi di Milano and Fondazione IRCCS Ca’ Granda Ospedale Maggiore Policlinico, Milan, Italy; Department of Clinical Sciences and Community Health, Valentina Bollati, Università degli Studi di Milano, Via San Barnaba 8, 20122 Milan, Italy

**Keywords:** Multiple sclerosis, Hypermethylation, DNA methylation, Repetitive elements, Epigenetics, Expanded disability status scale

## Abstract

**Background:**

Multiple sclerosis (MS) is a complex disorder of the central nervous system whose cause is currently unknown. Evidence is increasing that DNA methylation alterations could be involved in inflammatory and neurodegenerative diseases and could contribute to MS pathogenesis. Repetitive elements *Alu, LINE-1* and *SAT-*α, are widely known as estimators of global DNA methylation. We investigated *Alu, LINE-1* and *SAT-*α methylation levels to evaluate their difference in a case–control setup and their role as a marker of disability.

**Results:**

We obtained blood samples from 51 MS patients and 137 healthy volunteers matched by gender, age and smoking. Methylation was assessed using bisulfite-PCR-pyrosequencing. For all participants, medical history, physical and neurological examinations and screening laboratory tests were collected. All repetitive elements were hypermethylated in MS patients compared to healthy controls. A lower Expanded Disability Status Scale (EDSS) score was associated with a lower levels of *LINE-1* methylation for ‘EDSS = 1.0’ and ‘1.5 ≤ EDSS ≤ 2.5’ compared to an EDSS higher than 3, while *Alu* was associated with a higher level of methylation in these groups: ‘EDSS = 1.0’ and ‘1.5 ≤ EDSS ≤ 2.5’.

**Conclusions:**

MS patients exhibit an hypermethylation in repetitive elements compared to healthy controls. *Alu* and *LINE-1* were associated with degree of EDSS score. Forthcoming studies focusing on epigenetics and the multifactorial pathogenetic mechanism of MS could elucidate these links further.

**Electronic supplementary material:**

The online version of this article (doi:10.1186/s12863-016-0395-0) contains supplementary material, which is available to authorized users.

## Background

Multiple Sclerosis (MS) is a neurodegenerative disease involving the central nervous system (CNS) in which the infiltration of focal lymphocytes in myelin causes inflammatory lesions leading to axonal damage [1]. MS knows many different disease courses, of which relapsing-remitting is the most common one. This course is typed with attacks of worsening neurological functioning (relapsing), followed by partial or complete improvement of the symptoms (remitting) [2]. Women are more prone to develop the disease than men, and the age of onset of the patient ranges mostly between 20 and 40 years, with a transition to the progressive forms at the age of 40 to 50 [3].

The causal factors of MS are still poorly understood, but they are probably heterogeneous [4]. Recent findings suggest that the interplay between individual genetic susceptibility and external, environmental influences modulate the disease presentation and therapeutic responsiveness [5, 6]. Evidence is increasing that epigenetic mechanisms could be involved in inflammatory and neurodegenerative diseases and some studies have suggested that changes in these mechanisms could contribute to MS pathogenesis, representing a bridge between genetics and environmental causal factors [7]. Epigenetics are stable and heritable patterns that modify the phenotype without altering the genotype. In particular, DNA methylation has been the most extensively studied epigenetic marker. It involves adding a methyl group to the 5′ cytosine located in a CpG site to form 5 methylcytosine (5mC). A recent study from Huynh et al. found that genes important in oligodendrocyte survival were hypermethylated and had a lower expression in MS-affected human brain tissue compared to controls [8]. Furthermore, studies have shown a relation between repetitive elements hypermethylation and adverse health outcomes [9, 10].

Repetitive elements comprise roughly 66–69 % of the human genome [11]. It is estimated that one million *Alu* repeats are present in the human genome, which accounts for over 10 % of the entire genome [12], while 20 % are long interspersed nuclear element 1 (*LINE-1*) repeats [13] and 3–5 % comprises of alpha satellite DNA sequences (*SAT-*α) [14]. *SAT-*α sequences can be found in centromeres or centromere-adjacent heterochromatin containing several CpG sites [15]. The methylation status of these sequences might be considered a good estimate for global DNA methylation levels, and have been previously investigated in relation to human diseases [16, 17]. Hitherto there is limited information about *Alu, LINE-1* and *SAT-*α methylation in MS. Consequently, this study aims to evaluate the changes in methylation of these repetitive elements using a quantitative approach.

In the present study, we estimated repetitive element methylation levels in a population of 51 MS patients and 137 healthy controls matched for gender, age, and smoking status. These matching phenotypes were selected as they have been linked with differential methylation [18]. Methylation of *Alu, LINE-1* and *SAT-*α was evaluated in association with MS course markers (i.e. Multisystem Deficits at disease onset, presence of oligoclonal bands in cerebrospinal fluid, CSF) and Expanded Disability Status Scale (EDSS). Quantitative bisulfite-polymerase chain reaction (PCR)-pyrosequencing was applied to determine methylation levels of *Alu, LINE-1* and *SAT-*α.

## Methods

### Study design

Adult patients with MS (*n* = 51) were recruited at the Dino Ferrari Center, Fondazione Ca’ Granda, IRCCS Ospedale Maggiore Policlinico in Milan, from January to December 2010. Clinical diagnosis was performed using the McDonald criteria and their subsequent revisions [19–21]. All patients received standard clinical examinations, including medical history check, physical and neurological examinations, screening laboratory tests and a brain magnetic resonance imaging (MRI). Healthy control subjects (*n* = 137), matched for age, gender, smoking habits and ethnic background, were enrolled at the Department of Preventive Medicine, IRCCS Ospedale Maggiore Policlinico in Milan. Written informed consent was signed by each participant.

Healthy volunteers donated a blood sample at time of recruitment, while the MS patients donated blood at least one month after the completion of their steroid treatment following neurological symptoms. MS patients in remitting phase donated a single blood sample, while patients that were in the relapse phase during the first collection donated a second blood sample one month after an acute phase. Genomic DNA from 3 ml whole-blood was extracted using a FlexiGene DNA Kit (Qiagen Inc., Hilden, Germany), following manufacturer’s instructions. The DNA concentration in each sample was determined measuring the optical density (OD) at 260 nm wavelength on a NanoDrop 1000 Spectrophotometer (Thermo Fisher Scientific, Waltham, MA, USA). DNA samples were aliquoted and stored at −20 °C until further measurements.

### Analysis of DNA methylation

DNA methylation was quantified using bisulfite-PCR and pyrosequencing. In short, each sample (concentration 50 ng/μl) was treated using the EZ DNA Methylation Gold™ kit (Zymo Research, Orange, CA, USA). Final elution was performed in 30 μl of M-Elution Buffer. Bisulfite treated samples were used to assess DNA methylation of repetitive elements according to Yang et al. [16]. PCR primers were designed towards a consensus *Alu, LINE-1,* and *SAT-*α, and allowed the amplification of a representative pool of repetitive elements as a surrogate for global DNA methylation changes. PCR was carried out in 50 μl of Go-Taq Green Master Mix (Promega, Madison, WI, USA) with 1 pmol of the forward primer, 1 pmol of the reverse primer, 50 ng of bisulfite-treated DNA and water. Per primer pair (Table 1) either the forward or reverse primer was biotin labelled to purify the final PCR product by binding them to Streptavidin Sepharose HP beads (Amersham Biosciences, Uppsala, Sweden). The beads containing the bound PCR product were purified using the Pyrosequencing Vacuum Prep Tool (Pyrosequencing Inc., Westborough, MA, USA). The degree of methylation (%5mC) for each DNA locus is reported as a percentage of methylated cytosines divided by the sum of methylated and unmethylated cytosines. Samples were tested in triplicate for each marker to confirm reproducibility of the results. Concordance correlation coefficient obtained from duplicate runs was 0.518, 0.349 and 0.473 for *Alu, LINE-1* and *SAT-*α respectively.

**Table 1:** Sequences of the primers for *Alu, LINE-1* and *SAT-*α from left to right are from 5′ to 3′. Biotinilation (BIO) of the primers occurred at the 5′ end. Forward and reverse primers were utilized in the PCR step while the sequencing primer was used during pyrosequencing

Primer (5′ to 3′)	*Alu*	*LINE-1*	*SAT-*α
Forward	BIO-TTTTTATTAAAAATATAAAAATT	TTTTGAGTTAGGTGTGGGATATA	BIO-TGGATATTTGGATTATTGG
Reverse	CCCAAACTAAAATACAATAA	BIO-AAAATCAAAAAATTCCCTTTC	TTTCCAAAAAAATCTTCAAAAAAAT
Sequencing	AATAACTAAAATTACAAAC	AGTTAGGTGTGGGATATAGT	CTCAAAAATTTCTAAAAATACTTCTC

### Statistical analysis

A descriptive analysis was performed on demographic and clinical characteristics. Mean and standard deviation were reported for continuous variables, count and percentage for categorical variables. Mean age and mean levels of methylation markers: *Alu, LINE-1* and *SAT-*α were compared between MS patients and healthy controls by means of t-test.

Characteristics of MS patients and healthy controls were compared using the Chi-Square test or Fisher’s exact test for categorical variables and for continuous variables by the t-test. Correlation between three methylation markers in all participants was assessed with the Pearson correlation coefficient test. The same test was used to evaluate this correlation in controls and in MS patients. We computed odds ratios (ORs) and 95 % confidence intervals (CI) for the association of methylation levels with case/control status using multivariable logistic regression models, adjusted for age, gender and smoking status.

In order to investigate the potential association between disability status and the repetitive elements in MS patients, study participants were distributed equally between three groups according to their EDSS score: ‘EDSS = 1.0’, ‘1.5 ≤ EDSS ≤ 2.5’ and ‘3.0 ≤ EDSS ≤ 7.5’. Adjusted multivariable linear regression analyses was carried out to evaluate the relationship between the level of methylation in repetitive elements and EDSS score. The assumptions underlying the linear regression model (i.e. linearity, normality and homoscedasticity) were satisfied for all the independent continuous variables. Coefficients (ß) and 95 % CI were calculated firstly using ‘3.0 ≤ EDSS ≤ 7.5’ as reference for the other two groups and secondly using ‘EDSS = 1.0’ as the reference. Adjusted means and 95 % CI were also calculated and compared for each methylation marker.

Linear regression (adjusted for sex, age and smoking status) was applied to investigate the relationship between methylation marker level and disease activity, year of neurological symptoms onset, multisystem disorder, presence of oligoclonal bands, presence of multiple bands in CSF and spinal cord relapse. Statistical analysis was carried out using SAS software (version 9.2, SAS Institute, Milan, Italy). A two-sided *p*-value of less than 0.05 was considered statistically significant.

## Results

### Characteristics of the study population

Table 2 shows the characteristics of the 51 MS patients and 137 healthy control subjects. The mean age of the patients and control group was 39.6 and 41.4 years, respectively. Both groups had more female participants than male (25.5 % and 27.0 % for the patients and controls, respectively) while most of the subjects declared to be non-smokers (62.5 % of the MS patients and 62.0 % of the control group). Among MS patients, 58.8 % have had only one episode of neurological symptoms, while 41.2 % had at least two episodes. Thirty-eight percent of the patients had an EDSS score of 1 (38 %), 34 % a score between 1.5 and 2.5, and 30.0 % had a severe EDSS score of 3.5 to 7.

**Table 2:** Characteristics of Multiple Sclerosis (MS) patients and healthy controls

Characteristics	MS patients	Healthy controls	*p*-value
	*n* = 51	*n* = 137	
Age (years)			
Mean ± SD	39.6 ± 8.1	41.4 ± 9.1	>0.1
Gender			
Female	38 (74.5)	100 (73.0)	>0.1
Male	13 (25.5)	37 (27.0)	-
Smoking habit^a^			
Yes	15 (35.3)	52 (38.0)	>0.1
No	33 (64.7)	85 (62.0)	-
Neurological episodes			
Only one	30 (58.8)	-	
At least two	21 (41.2)	-	
Disability^b^			
EDSS = 1.0	18 (36.0)	-	
1.5 ≤ EDSS ≤ 2.5	17 (34.0)	-	
3.0 ≤ EDSS ≤ 7	15 (30.0)	-	
Onset of MS			
1981 – 1999	18 (35.3)	-	
2000 – 2004	15 (29.4)	-	
2005 – 2010	18 (35.3)	-	
Multisystem deficits at onset			
Yes	14 (27.5)	-	
No	37 (72.5)	-	
Oligoclonal Bands in CSF^c^			
Yes	44 (89.8)	-	
No	5 (10.2)	-	
Methylation markers (%5mC)			
* Alu* ^*d*^	25.3 ± 0.6	24.5 ± 1.1	<0.001
* LINE-1*	85.1 ± 1.4	82.6 ± 2.8	<0.001
* SAT- α*	80.3 ± 2.6	78.9 ± 2.8	0.014

Data is presented as Mean ± SD or number (%). Disability is defined as ‘expanded disability status scale’ (EDSS)

Statistical comparison: student t-test between MS patients versus healthy controls: age and methylation markers. Chi-Square, total population: gender (50 males versus 138 females) and smoking habit (67 smokers versus 118 non-smokers)

^a^Data available for: 48, ^b^50 and ^c^49 MS patients and ^d^135 healthy controls

### Internal correlations of repetitive elements

**Fig. 1:**
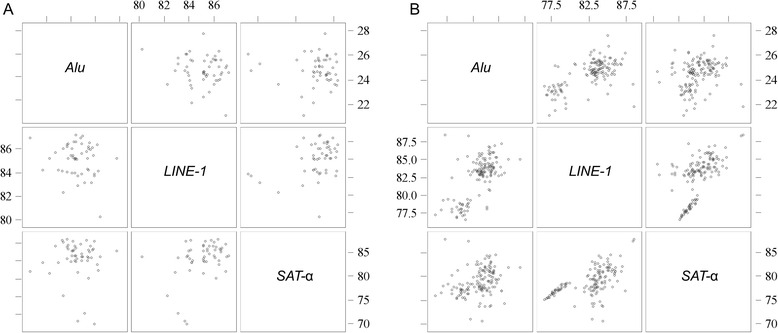
Scatter plot comparing *Alu*, *LINE-1* and *SAT-*α methylation levels. In panel **a**, the correlation is represented for the MS patients, in panel **b** for the healthy controls. For the latter group, all repetitive elements were significantly correlated with each other in terms of methylation (*Alu* and *LINE-1,* ρ = 0.627; *Alu* and *SAT-*α, ρ = 0.253; *LINE-1* and *SAT-*α, ρ = 0.559). In the MS patients, only *LINE-1* methylation was significantly correlated with *SAT-*α methylation (ρ = 0.380). Significance level was set at 0.05

Considering the entire study population, *Alu* methylation level was positively correlated with the methylation level of both *LINE-1* (ρ = 0.614, *p* < 0.0001) and *SAT-*α (ρ = 0.259, *p* = 0.0003). *LINE-1* methylation was also positively correlated with *SAT*-α (ρ = 0.553, *p* < 0.0001). After case/control stratification, these correlations were confirmed in the control group (*Alu* and *LINE-1:* ρ = 0.627, *p* < 0.0001; *Alu* and *SAT-*α: ρ = 0.253, *p* = 0.0003; *LINE-1* and *SAT*-α: ρ = 0.559, *p* < 0.0001). Conversely, in the MS patients group (Fig. 1), only *LINE-1* methylation was correlated with the degree of methylation in *SAT*-α (ρ = 0.380, *p* < 0.0059). *Alu* methylation levels were not correlated with the methylation of either *LINE-1* (ρ = −0.015, *p* = 0.285) or *SAT*-α (ρ = −0.025, *p* = 0.861).

### Differences in methylation levels in MS patients and healthy control subjects

As shown in Table 1, all methylation markers showed a significant increase in methylation in the MS patients group compared to the healthy control group. *Alu* methylation was 25.3 % 5mC in MS patients and 24.5 % 5mC in healthy controls (*p* < 0.0001). *LINE-1* methylation was 85.1 %5mC in MS patients and 82.6 % 5mC in the healthy controls (*p <* 0.0001). *SAT-*α methylation was 80.3 % 5mC in MS patients and 78.9 %5mC in healthy controls (*p* < 0.003). The difference between cases and controls remained significant after adjusting for age, gender and smoking status in a multivariate logistic regression analysis for all markers (*Alu: p =* 0.0029; *LINE-1: p* = 0.0003; *SAT-*α: *p* = 0.0456), respectively the OR for *Alu*, *LINE-1* and *SAT-*α were 2.137, 1.619 and 1.155. CpG site specific analyses are shown in Additional file 1: Figure S1 and their characteristics are presented in Additional file 2: Table S1.

### Methylation levels in EDSS in MS patients

**Fig. 2:**
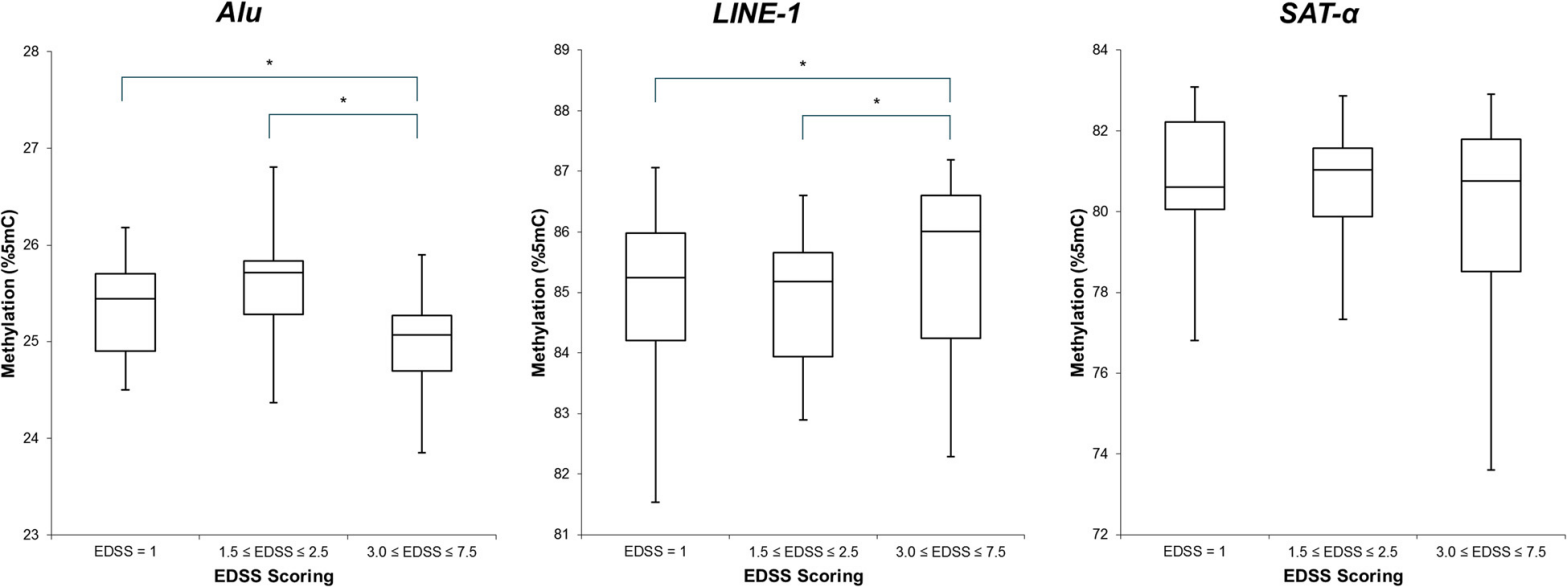
Methylation levels of *Alu, LINE-1* and *SAT-*α in MS patients, grouped by Expanded Disability Status Scale (EDSS), are represented as box plots. Data were adjusted for age, sex and smoking habit. All groups had a similar number of individuals: 18 participants for ‘EDSS = 1.0’, 17 for ‘1.5 ≤ EDSS ≤ 2.5’ and 15 for ‘3.0 ≤ EDSS ≤ 7.5’. For both *Alu* and *LINE-1* the highest EDSS group (the reference) was significantly different from the lower two groups. An absolute difference in methylation of 0.47 %5mC can be observed for ‘EDSS = 1’ (*p* = 0.034) and 0.63 %5mC for ‘1.5 ≤ EDSS ≤ 2.5’ (*p* = 0.007) compared with the reference group for *Alu*. In *LINE-1*, methylation decreased with 0.95 %5mC for ‘EDSS = 1’ (*p* = 0.050) and 1.07 % for ‘1.5 ≤ EDSS ≤ 2.5’ (*p* = 0.033) compared to the reference group. * *p* < 0.05

When we compared EDSS classes, using the highest EDSS class as reference, *Alu* methylation was elevated in both ‘EDSS = 1.0’ (ß = 0.47; 95 % CI = 0.04 to 0.90; *p* = 0.034) and ‘1.5 ≤ EDSS ≤ 2.5’ (ß = 0.63; 95 % CI = 0.18 to 1.08; *p* = 0.007). *LINE-1* methylation was lower in both ‘EDSS = 1.0’ (ß = −0.95; 95 % CI = −1.89 to 0.003; *p* = 0.050) and ‘1.5 ≤ EDSS ≤ 2.5’ (ß = −1.07; 95 % CI = −2.06 to −0.09; *p* = 0.033). *SAT-*α methylation was not significantly different in the EDSS groups. Results are shown in Fig. 2.

### Methylation levels and MS course

No significant differences were observed in the methylation markers between subjects who experienced just one neurological episode or at least two episodes. There was no significant association between methylation and years of onset. Furthermore, no differences were observed between methylation and multisystem disorders. The presence or amount of oligoclonal bands in CSF was also not associated with repetitive element methylation. Finally, no significant differences were found between patients with and without spinal cord relapse. Data is presented in Additional file 3: Table S2.

## Discussion

In the present study, we evaluated methylation levels of *Alu, LINE-1* and *SAT-*α in 51 MS patients and 137 healthy volunteers. MS patients showed hypermethylation in all repetitive elements compared to healthy controls. Furthermore, the correlations between these global methylation markers in the control group were lost in MS patients. Moreover, we demonstrated that worsening disability score was associated with hypomethylation in *Alu* and hypermethylation in *LINE-1*. These findings suggest that MS patients have an altered methylation of repetitive elements in blood leukocytes compared to healthy individuals. To our knowledge, this is the first study to investigate associations between methylation of *Alu, LINE-1* and *SAT-*α, and MS.

Previous research found associations of global DNA hypermethylation with neurodegenerative and neurological disorders like Alzheimer’s disease [22, 23] and post-traumatic stress disorder [24]. However, the pathogenesis for MS is different from these disorders. Furthermore, a recent case–control study concerning genome-wide DNA methylation in MS patients finds immune cells to experience hypermethylation [25]. A second epigenetic mechanism involved in MS are micro RNAs (miRNA). These miRNAs are involved in gene silencing by degrading target mRNA sequences to prevent their translation into proteins. Literature suggest that certain miRNA (e.g. miR-155 and miR-326) are highly upregulated in active MS lesions [26]. This upregulation can in turn lead to macrophage activation, myelin degradation and could drive the MS progression [7, 27]. Furthermore, dysregulation of miR-155 and miR-326 has been observed in peripheral blood mononuclear cells and CD4+ T cells of MS patients respectively [7]. We found all repetitive elements to be hypermethylated in MS patients compared to the healthy controls. Since these repetitive elements are a marker for global DNA methylation levels, our results suggest that in MS the genome exhibits a higher degree of methylation. While a hypermethylation would indicate a downregulation of miR-155 and miR-326, no hypermethylation was observed in CD4+ T cells of MS patients, only in CD8+ T cells [28].

Healthy controls showed significant correlations among the degree of methylation for all repetitive elements, while in patients with MS only *LINE-1* and *SAT-*α methylation levels remained significantly correlated. We speculate that for healthy subjects the upkeep mechanisms for these elements are able to maintain the normal methylation levels, while in MS patients these upkeep regulations might be partially lost. This could in turn lead to an abnormal maintenance of repetitive element methylation levels causing their reciprocal correlations to diminish.

We observed different methylation levels in *Alu* and *LINE-1* among the three different EDSS groups. Methylation of *Alu* decreased with increasing EDSS scores. Although this tendency was also observed in *SAT-*α, it was not significant. In contrast, *LINE-1* methylation was positively associated with EDSS score. These results could be counterintuitive as both *Alu* and *LINE-1* are markers of global DNA methylation and are positively correlated with each other. However, as mentioned earlier, in MS patients this correlation disappears and they tend to be inversely (but not significantly) associated. Prior studies find that EDSS value is strongly correlated with axonal damage and neurodegeneration [29, 30]. Furthermore, a possible mechanism for MS progression and axonal loss could be via oxidative stress, caused by a decrease in antioxidant levels, which might lead to DNA damage [31]. A possible mode of action is through DNA methyl-transferase (DNMT), which are recruited to sites of DNA damage and have previously been suggested as being directly involved in DNA damage repair [32]. These DNMTs have been reported as important and essential elements in development and are responsible for genomic integrity due to their methylating capabilities [32, 33]. As Bollati et al. proposed, a *LINE-1* hypermethylation could be the consequence of this DNMT upregulation [22]. The effect of DNMT overexpression was observed in brain tissue of mice, where they associated this upregulation with an increase in methylation in motor neuron cells [34].

Although we found MS patients to have hypermethylated repetitive elements, no distinct methylation differences were observed in different clinical MS groups (i.e. disease activity, phase of MS, days since relapse, year of onset, multisystem disorder, spinal cord relapse and the presence of oligoclonal bands in CSF). Our data had a limited amount of MS patients in different clinical groups, which might contribute to non-significant results.

## Conclusion

As MS has a multifactorial pathology, hypotheses focusing solely on environmental or genetic components are missing key components. However, epigenetics are able to bridge the gap between these theories and appears to be promising. In summary, we found that 1) *Alu, LINE-1* and *SAT-*α repetitive elements were hypermethylated in MS patients, 2) *Alu, LINE-1* and *SAT-*α are positively correlated with each other in healthy controls, while only *LINE-1* and *SAT-*α are correlated in MS patients and 3) EDSS values were associated with differential methylation in *Alu* and *LINE-1* elements. We suggest that forthcoming investigations should include a higher number of MS patients to increase statistical power. Future studies focusing on epigenetics and both disease course and clinical prognostic markers could further elucidate the understanding of the multifactorial pathology of MS.

## Abbreviations

5mC, 5-methylcytosine; CSF, cerebrospinal fluid; DNMT, DNA methyl-transferase; EDSS, expanded disability status scale; LINE-1, long interspersed nuclear elements; MS, multiple sclerosis; OR, odds ratio; PCR, polymerase chain reaction; SAT-a, satellite DNA alpha

## Additional files

Additional file 1: Figure S1. CpG specific odds ratios were calculated between MS (*n* = 51) patients and Healthy controls (*n* = 137 for *LINE-1* and *SAT-*α; *n* = 135 for *Alu*). Methylation was assessed in 3 CpG sites for *Alu* and *SAT-*α and 4 CpG sites in *LINE-1*. Estimates are presented as odds ratios, adjusted for age, gender and smoking status, and were calculated using the multivariate logistic regression analysis. (DOCX 564 kb) Additional file 2: Table S1. Characteristics of Multiple Sclerosis (MS) patients and healthy controls for each methylation marker. Methylation is subdivided as ‘mean’ (i.e. average of the separate positions) and the individual positions of the markers. (DOCX 66 kb) Additional file 3: Table S2. Not significant data is presented as the estimate (ß) and their respective p-value (*p*). Disease activity is presented as ‘annualized relapse rate’ (ARR). The presence and amount of oligoclonal bands was measured in cerebrospinal fluid (CSF). (DOCX 18 kb)
