# Supplementary material for: Repetitive element hypermethylation in multiple sclerosis patients
Source: BMC Genet. 2016 Jun 18;17:84. doi: 10.1186/s12863-016-0395-0 (PMC4912727; doi:10.1186/s12863-016-0395-0)
Supplement: Additional file 1: Figure S1. — CpG specific odds ratios were calculated between MS (n = 51) patients and Healthy controls (n = 137 for LINE-1 and SAT-α; n = 135 for Alu). Methylation was assessed in 3 CpG sites for Alu and SAT-α and 4 CpG sites in LINE-1. Estimates are presented as odds ratios, adjusted for age, gender and smoking status, and were calculated using the multivariate logistic regression analysis. (DOCX 564 kb) [file 12863_2016_395_MOESM1_ESM.docx]

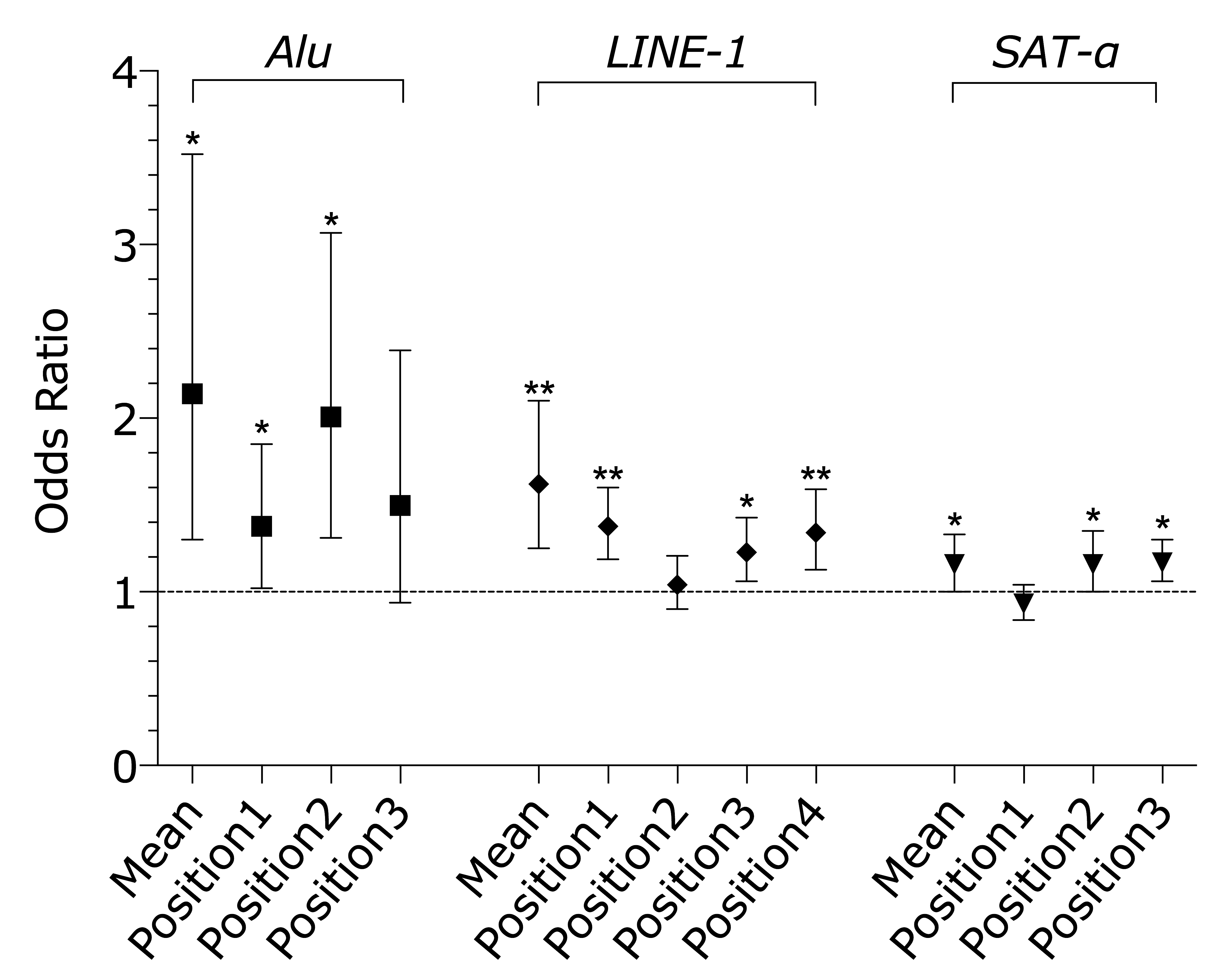


**Supplementary Figure 1.** CpG specific odds ratios were calculated between MS (n = 51) patients and Healthy controls (n = 137 for *LINE-1* and *SAT-*α; n = 135 for *Alu*). Methylation was assessed in 3 CpG sites for *Alu* and *SAT-*α and 4 CpG sites in *LINE-1*. Estimates are presented as odds ratios, adjusted for age, gender and smoking status, and were calculated using the multivariate logistic regression analysis. * *p* < 0.05; ** *p* < 0.001
