# Supplementary material for: Repetitive element hypermethylation in multiple sclerosis patients
Source: BMC Genet. 2016 Jun 18;17:84. doi: 10.1186/s12863-016-0395-0 (PMC4912727; doi:10.1186/s12863-016-0395-0)
Supplement: Additional file 2: Table S1. — Characteristics of Multiple Sclerosis (MS) patients and healthy controls for each methylation marker. Methylation is subdivided as ‘mean’ (i.e. average of the separate positions) and the individual positions of the markers. (DOCX 66 kb) [file 12863_2016_395_MOESM2_ESM.docx]

| **Supplementary Table 1**. Characteristics of Multiple Sclerosis (MS) patients and healthy controls for each methylation marker. Methylation is subdivided as ‘mean’ (i.e. average of the separate positions) and the individual positions of the markers. Data is presented as Mean ± SD. | | | |
| --- | --- | --- | --- |
| **Characteristics** | **MS Patients n = 51** | **Healthy Controls n = 137** | **p-value** |
| **Methylation markers (%5mC)** |  |  |  |
| ***Alu^a^*** |  |  |  |
| Mean | 25.3 ± 0.6 | 24.5 ± 1.1 | < 0.001 |
| Position 1 | 34.3 ± 1.2 | 33.7 ± 1.5 | 0.005 |
| Position 2 | 25.0 ± 0.8 | 23.9 ± 1.6 | < 0.001 |
| Position 3 | 16.5 ± 16.3 | 15.9 ± 0.9 | < 0.001 |
| ***LINE-1*** |  |  |  |
| Mean | 85.1 ± 1.4 | 82.6 ± 2.8 | < 0.001 |
| Position 1 | 87.9 ± 2.5 | 83.8 ± 3.9 | < 0.001 |
| Position 2 | 81.2 ± 2.0 | 80.0 ± 3.1 | 0.008 |
| Position 3 | 83.5 ± 2.8 | 81.0 ± 3.0 | < 0.001 |
| Position 4 | 87.8 ± 2.6 | 85.7 ± 2.9 | < 0.001 |
| ***SAT-α*** |  |  |  |
| Mean | 80.3 ± 2.6 | 78.9 ± 2.8 | 0.014 |
| Position 1 | 75.4 ± 3.0 | 76.8 ± 3.7 | 0.024 |
| Position 2 | 78.9 ± 2.0 | 77.7 ± 2.9 | 0.002 |
| Position 3 | 86.5 ± 5.0 | 82.2 ± 5.5 | 0.014 |
| Statistical comparison: student t-test between MS patients versus healthy controls.  ^a^ Data available for: 135 healthy controls | | | |
