# Supplementary material for: Repetitive element hypermethylation in multiple sclerosis patients
Source: BMC Genet. 2016 Jun 18;17:84. doi: 10.1186/s12863-016-0395-0 (PMC4912727; doi:10.1186/s12863-016-0395-0)
Supplement: Additional file 3: Table S2. — Not significant data is presented as the estimate (ß) and their respective p-value (p). Disease activity is presented as ‘annualized relapse rate’ (ARR). The presence and amount of oligoclonal bands was measured in cerebrospinal fluid (CSF). (DOCX 18 kb) [file 12863_2016_395_MOESM3_ESM.docx]

| **Supplementary Table 2.** Not significant data is presented as the estimate (ß) and their respective p-value (*p*). Disease activity is presented as ‘annualized relapse rate’ (ARR). The presence and amount of oligoclonal bands was measured in cerebrospinal fluid (CSF). | | | | | | | | | | |
| --- | --- | --- | --- | --- | --- | --- | --- | --- | --- | --- |
|  |  |  | ***Alu*** | |  | ***LINE-1*** | |  | ***SAT-*α** | |
| **Type** | **n** |  | **ß** | ***p*** |  | **ß** | ***p*** |  | **ß** | ***p*** |
| **Neurological episodes** |  |  |  |  |  |  |  |  |  |  |
| Only one | 29 |  | 0.094 | 0.64 |  | 0.079 | 0.86 |  | -0.25 | 0.74 |
| At least two | 16 |  | *Ref.* | *.* |  | *Ref.* | *.* |  | *Ref.* | . |
| **Year of onset** |  |  |  |  |  |  |  |  |  |  |
| 1981 – 1999 | 18 |  | -0.19 | 0.47 |  | 0.31 | 0.58 |  | 0.52 | 0.57 |
| 2000 – 2004 | 15 |  | 0.27 | 0.25 |  | -0.74 | 0.13 |  | -0.35 | 0.67 |
| 2005 – 2010 | 18 |  | *Ref.* | *.* |  | *Ref.* | *.* |  | *Ref.* | *.* |
| **Multisystem disorder** |  |  |  |  |  |  |  |  |  |  |
| No | 37 |  | -0.17 | 0.43 |  | 0.027 | 0.96 |  | 0.44 | 0.57 |
| Yes | 14 |  | *Ref.* | *.* |  | *Ref.* | *.* |  | *Ref.* | *.* |
| **Oligoclonal bands in CSF** |  |  |  |  |  |  |  |  |  |  |
| No | 5 |  | -0.21 | 0.43 |  | -0.14 | 0.84 |  | -1.13 | 0.28 |
| Yes | 44 |  | *Ref.* | *.* |  | *Ref.* | *.* |  | *Ref.* | *.* |
| **Multiple bands in CSF** |  |  |  |  |  |  |  |  |  |  |
| No | 37 |  | 0.14 | 0.47 |  | 0.090 | 0.85 |  | -0.63 | 0.41 |
| Yes | 12 |  | *Ref.* | *.* |  | *Ref.* | *.* |  | *Ref.* | *.* |
| **Spinal cord relapse** |  |  |  |  |  |  |  |  |  |  |
| No | 15 |  | 0.26 | 0.54 |  | -0.25 | 0.69 |  | -2.03 | 0.19 |
| Yes | 5 |  | *Ref.* | *.* |  | *Ref.* | *.* |  | *Ref.* | *.* |
|  | | | | | | | | | | |
